# Establishment of a Monoclonal Antibody-Based Enzyme-Linked Immunosorbent Assay to Measure Soluble B7-H5 in Patients with Cancer

**DOI:** 10.1155/2022/3013185

**Published:** 2022-08-04

**Authors:** Tongguo Shi, Shuru Zhou, Ting Zhang, Shiyang Han, Li Zhang, Fengqing Fu, Ruhong Yan, Xueguang Zhang

**Affiliations:** ^1^Jiangsu Institute of Clinical Immunology, The First Affiliated Hospital of Soochow University, Suzhou, 215000 Jiangsu, China; ^2^The Aoyang Cancer Institute, Affiliated Aoyang Hospital of Jiangsu University, Zhangjiagang, 215600 Jiangsu, China; ^3^Department of Oncology, The First Affiliated Hospital of Soochow University, Suzhou, 215000 Jiangsu, China; ^4^Soochow University-Bright Scistar Antibody Joint Laboratory, Suzhou, 215000 Jiangsu, China; ^5^State Key Laboratory of Radiation Medicine and Protection, Soochow University, Suzhou, 215123 Jiangsu, China; ^6^Department of Clinical Laboratory, Suzhou Science & Technology Town Hospital, Suzhou, 215153 Jiangsu, China

## Abstract

B7-H5, an immune checkpoint molecule, is markedly upregulated in multiple cancers and plays an important role in tumor progression and immune escape. However, the expression and significance of soluble B7-H5 (sB7-H5) in cancer remain unclear. Herein, we generated two novel mouse anti-human B7-H5 monoclonal antibodies (mAbs) 2E5 and 7B10, which had different epitopes. Based on the two mAbs, a sandwich enzyme-linked immunosorbent assay (ELISA) system was developed. Using this ELISA, we found that compared with healthy controls (HCs), sB7-H5 levels were significantly increased in the serum of patients with gastric cancer (GC), colorectal cancer (CRC), and lung cancer (LC) and were associated with TNM stage and metastasis. Receiver operating characteristic (ROC) curve analysis showed that sB7-H5 has diagnostic value for GC, CRC, and LC. Collectively, our findings delineate that sB7-H5 may be used as a predictor for diagnosis of cancer and a potential therapeutic target for cancer treatment.

## 1. Introduction

Cancer immunotherapy based on immune checkpoint inhibitors (ICIs) has achieved encouraging results in multiple malignancies, such as melanoma and lung cancer [1–3]. To date, ICIs targeting PD-1, PD-L1, and CTLA-4 have been approved by the US Food and Drug Administration (FDA) for use in the treatment of human tumors [3]. However, only a small percentage of patients with cancer respond to these ICIs [4]. Therefore, it is needed to discover new inhibitory checkpoints and their target molecules and to enrich existing ICI therapy.

B7-H5, also designated as VISTA and PD-1H, is a member of the immune checkpoints of the B7 family [5–7]. As a type I transmembrane protein, B7-H5 shares homology to PD-1, CD28, and CTLA-4 [5, 8]. Many studies showed that the protein levels of B7-H5 were markedly upregulated in the tissue specimens of multiple cancers such as gastric cancer (GC), colorectal cancer (CRC), and lung cancer (LC) [7, 9, 10]. Furthermore, aberrant B7-H5 expression was associated with the clinicopathological features and prognosis of tumor patients [11–13]. It has been confirmed that human B7-H5 owns two binding partners with immunosuppressive functions, PSGL-1 and VSIG3 [5], and exerts negative regulatory functions by suppressing the activation, proliferation, and cytokine secretion of T cells [14]. These findings indicated the important roles of B7-H5 in tumor progression and immune escape.

Recently, soluble forms of immune checkpoint molecules including PD-1, PD-L1, B7-H3, and B7-H4 in cancer have been demonstrated to be correlated with advanced stage, metastatic status, prognosis, and ICI treatment of patients with cancer [15, 16]. Similar to most immune checkpoint molecules, B7-H5 also has a soluble form and contributes to various diseases [17, 18]. In this study, we generated two novel mouse anti-human B7-H5 monoclonal antibodies (mAbs) 2E5 and 7B10 and established a sandwich enzyme-linked immunosorbent assay (ELISA) system based on the two mAbs with different epitopes. Using this ELISA, we demonstrated that soluble B7-H5 (sB7-H5) levels were significantly increased in the serum of patients with GC, CRC, and LC compared with healthy controls (HCs) and associated with tumor node metastasis (TNM) stage and metastasis. Moreover, the receiver operating characteristic (ROC) curve analysis showed that sB7-H5 has diagnostic value for GC, CRC, and LC. Herein, our findings suggest that sB7-H5 may be used as a predictor for diagnosis or immunotherapy of cancer.

## 2. Materials and Methods

### 2.1. Generation of Anti-Human B7-H5 Monoclonal Antibodies

Six to eight-week-old female BALB/c mice were obtained from the Animal Research Center of Soochow University. All animal experiments were approved by the Ethics Committee of Soochow University (approval number: SUDA20210918A02). B7-H5 mAb generation was performed as previously described [19, 20]. Briefly, 1 × 10^7^ B7-H5 overexpressing L929 cells (Bright Scistar Biotechnology Co., Ltd., Suzhou, China) pretreated with mitomycin were intraperitoneally administered to BALB/c mice four times every 21 days. Then, the splenocytes of immunized mice were harvested and used to fuse with SP2/0 cells in the presence of 50% polyethylene glycol (PEG). The fusion cells were cultured with Dulbecco's modified Eagle's medium (DMEM, Biological Industries, BeitHaemek, Israel) containing hypoxanthine-aminopterin-thymidine (HAT; Sigma, St Louis, MO, USA) and 15% fetal bovine serum (FBS, Gibco, Grand Island, New York, USA). To establish hybridoma cell lines, supernatants from mixed cell cultures were screened by flow cytometry. Finally, the protein G sepharose affinity column (GE Healthcare Biosciences AB, Uppsala, Sweden) was used to purify the ascite antibodies.

### 2.2. Characterization of B7-H5 Monoclonal Antibodies

Mouse monoclonal antibody isotyping kit (Roche, Basel, Switzerland) was used to identify immunoglobulin (Ig) isotypes according to the manufacturer's protocol. To determine the binding ability of antibodies to recombinant human B7-H5 protein (R&D Systems, Minneapolis, MN, USA), indirect ELISA analysis was used. Briefly, a 96-well plate was coated with carbonate buffer solution (CBS) containing B7-H5 Ig overnight at 4°C. After 2 h of incubation with 3% bovine serum albumin (BSA, Beyotime, Shanghai, China), biotin-labeled B7-H5 mAbs were added to the 96-well plate and incubated at 37°C for 2 h. Then, the plate was stained with horseradish peroxidase- (HRP-) streptavidin (Sigma, St Louis, MO, USA) at 1 : 10000 for 1 h at 37°C and followed by addition with Tetramethylbenzidine (TMB; Sigma). After stopping with 2 M sulfuric acid, the absorbance at 450 nm was measured by a microplate reader (Bio-Rad, Hercules, CA, USA).

The competition assay based on ELISA was performed as previously described [20]. Briefly, unlabeled anti-B7-H5 mAb 2E5 or 7B10 in CBS was used to coat the 96-well plate overnight at 4°C. After blocking, recombinant human B7-H5 protein was added to the plate and incubated for 2 h at 37°C. Then, the plate was stained with biotin-labeled 7B10 or 2E5 for 1 h at 37°C and treated with TMB and sulfuric acid solution, successively. Finally, the absorbance at 450 nm was measured.

### 2.3. Western Blotting

To further analyze the binding capacity of 2E5 and 7B10 to human B7-H5, we performed western blot analysis. Recombinant human B7-H5 protein (10 *μ*g) was loaded on 12% nonreducing SDS-PAGE gels and transferred onto 0.45 *μ*m PVDF membranes (GE Healthcare Life Science, Freiburg, Germany). After blocking with 5% nonfat dry milk in PBS, the membrane was incubated with the following primary antibodies: anti-B7-H5 mAb 2E5, 7B10, or commercial B7-H5 antibody D1L2G (CST, Danvers, MA, USA) overnight at 4°C. Then, the membrane was incubated with HRP-conjugated secondary antibodies for 1 h at room temperature, followed by visualization with an ECL reagent (NCM Biotech, Suzhou, China) using a Chemi DocTM MP Imaging System (Bio-Rad).

### 2.4. Establishment of sB7-H5 ELISA Kit

The 96-well plate was coated with 2E5 (5 *μ*g/ml) in CBS overnight. The next day, the plate was blocked with 3% BSA and added with purified human B7-H5. After incubation for 2 h at 37°C, the plate was incubated with biotin-labeled 7B10 (0.25 *μ*g/ml) for 1 h at 37°C. Then, the plate was incubated with HRP-streptavidin, added with TMB, and followed by stopping with 2 M sulfuric acid. The absorbance at 450 nm was measured by the microplate reader.

### 2.5. Precision and Specificity Assay of sB7-H5 ELISA

We tested different concentrations of B7-H5-Fc fusion protein (600, 300, and 150 pg/ml) to assess the intra-assay and interassay precision 10 times. To evaluate the specificity, other homologous proteins including sB7-H1, sB7-H2, sB7-H3, sB7-H4, and sB7-H6 were detected by ELISA.

### 2.6. Patients and Specimens

This study was approved by the medical ethics committee of Affiliated Aoyang Hospital of Jiangsu University (approval number: 2021015), and informed consent was signed by each patient. Peripheral blood samples were collected from 256 patients, including 50 cases of GC, 74 cases of CRC, 122 cases of LC, and 109 individual HCs at Affiliated Aoyang Hospital of Jiangsu University (Zhangjiagang, China). Detailed clinicopathological information is provided in Tables 1–3. The serum specimens were separated by centrifugation and stored at -80°C. The level of sB7-H5 in serum specimens was detected by sB7-H5 ELISA.

### 2.7. Statistical Analysis

Statistical analyses were performed by GraphPad Prism 6.0 (La Jolla, CA, USA). The data of sB7-H5 were nonnormally distributed. The differences between groups were analyzed by the Mann-Whitney *U* test. The ROC curves with a calculation of the areas under the ROC curves (AUCs) were calculated. *P* < 0.05 was considered the significant difference.

## 3. Results

### 3.1. Two Novel Anti-Human B7-H5 mAbs Were Identified

**Figure 1:**
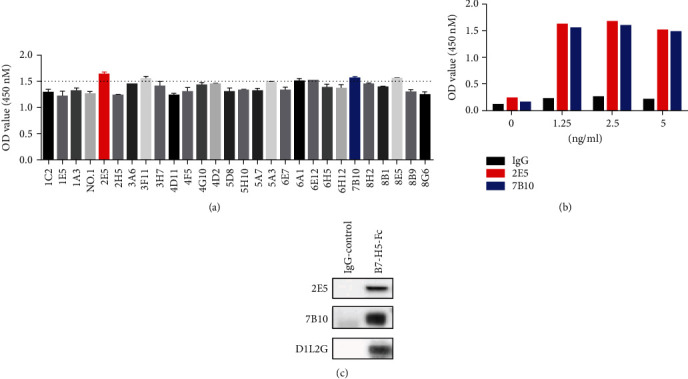
Characterization of B7-H5 monoclonal antibodies (mAbs). (a, b) Indirect ELISA was used to determine the binding ability of antibodies to immobilize B7-H4 Fc. (c) Western blot showed the antibodies recognized B7-H5 protein.

As shown in Figure 1(a), there were 28 clones of mouse anti-human B7-H5 mAbs, which showed positive binding by ELISA. Among these clones, 2E5 and 7B10 were chosen for further validation. The isotypes of 2E5 and 7B10 were IgG1 *κ*. The results of indirect ELISA showed that both 2E5 and 7B10 could bind to the immobilized B7-H5 Fc protein (Figure 1(b)). Additionally, Western blotting also showed that 2E5 and 7B10 recognized the target protein bands of B7-H5 Fc, but not IgG protein (Figure 1(c)).

**Figure 2:**
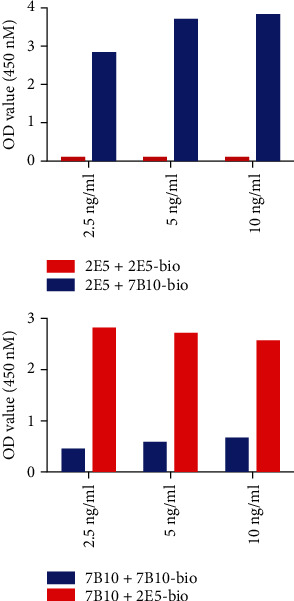
2E5 and 7B10 monoclonal antibodies (mAbs) recognized different epitopes. Mutual competition assay by ELISA.

To characterize the epitopes on B7-H5 that mediate 2E5 or 7B10 binding, we performed the competitive binding assay by ELISA. As shown in Figure 2, one unlabeled mAb did not inhibit the binding of the other biotin-labeled mAb. These results suggested that 2E5 and 7B10 recognized two different epitopes of B7-H5.

### 3.2. Establishment of a Novel Sandwich sB7-H5 ELISA

**Figure 3:**
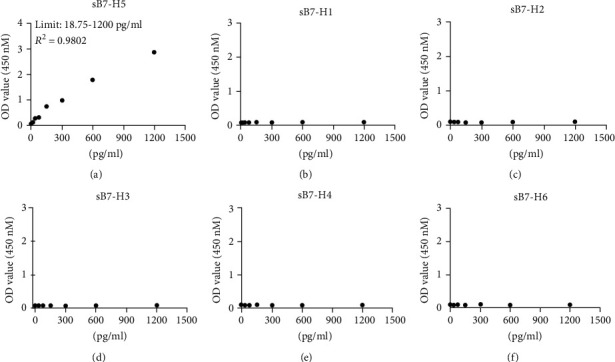
Establishment of sB7-H5 ELISA. (a) The unlabeled 2E5 was 5 *μ*g/ml, and the biotin-labeled 7B10 was 0.25 *μ*g/ml. The serial dilutions of the B7-H5 Ig protein starting from 1200 pg/ml were detected by ELISA. *R*^2^ represents the correlation coefficient. (b–f) The specificity of sB7-H5 ELISA. The proteins of sB7-H1, sB7-H2, sB7-H3, sB7-H4, and sB7-H6 were detected by ELISA.

To establish a novel sandwich sB7-H5 ELISA using 2E5 and 7B10, we optimized the working concentrations. A sensitive ELISA was established using 5 *μ*g/ml 2E5 as the coating antibody and 0.25 *μ*g/ml biotin-7B10 as the detecting antibody. As shown in Figure 3(a), the detectable limitation of B7-H5 Fc protein was 18.75–1200 pg/ml, with *R*^2^ being 0.9802. For the specificity analysis, the sB7-H5 ELISA was used to detect other homologous proteins including sB7-H1, sB7-H2, sB7-H3, sB7-H4, and sB7-H6. It did not cross-react with these homologous proteins (Figures 3(b)–3(f)). Moreover, the intra-assay and interassay precision of the sB7-H5 ELISA were evaluated, and the results showed that the ELISA was very moderate with little well-to-well and plate-to-plate variation (CV% < 10%, Table 4). Collectively, a high sensitivity sandwich sB7-H5 ELISA system was established.

### 3.3. sB7-H5 Levels Were Increased in the Serum of Patients with Gastric, Colorectal, and Lung Cancer

**Figure 4:**
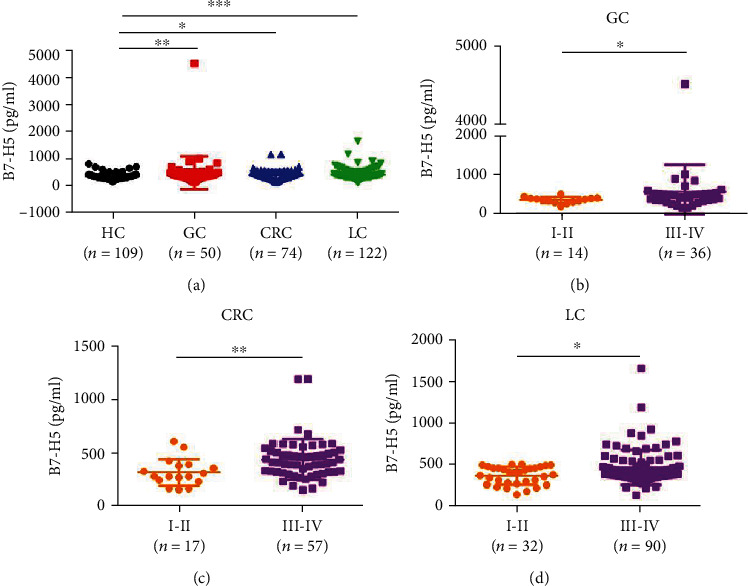
The levels of sB7-H5 in the serum of patients with gastric, colorectal, and lung cancer. (a) The sB7-H5 ELISA showed the concentration of sB7-H5 in the serum of GC (*n* = 50), CRC (*n* = 74), and LC (*n* = 122) patients. (b–d) The association between sB7-H5 levels and TNM stage. ^∗^*P* < 0.05, ^∗∗^*P* < 0.01, and ^∗∗∗^*P* < 0.001.

Next, we used the sB7-H5 ELISA system to investigate the levels of sB7-H5 in the serum of HCs and patients with GC, CRC, and LC. As shown in Figure 4(a), the concentration of sB7-H5 was markedly higher in GC, CRC, and LC patients when compared with HC.

### 3.4. The Associations between the Levels of sB7-H5 and Clinicopathologic Features of Gastric, Colorectal, and Lung Cancer Patients

We then attempted to analyze the associations between the levels of sB7-H5 in the sera of patients with cancer and the clinicopathologic features of the patients (Tables 1–3). As shown in Table 1 and Figure 4(b), sB7-H5 levels in the GC patients were significantly correlated with TNM staging (*P* < 0.05) and were not related with age, sex, or metastasis. Furthermore, the concentration of sB7-H5 in the CRC and LC patients was surely associated with TNM stage (*P* < 0.01 for CRC and *P* < 0.05 for LC) and metastasis (Figures 4(c) and 4(d) and Tables 2 and 3, *P* < 0.05 for CRC and *P* < 0.01 for LC).

### 3.5. Diagnostic Value of sB7-H5

**Figure 5:**
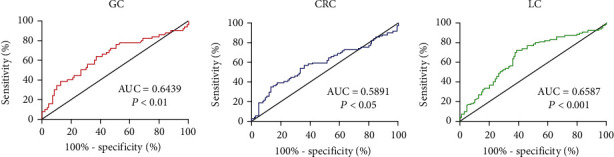
ROC curves of sB7-H5 in GC, CRC, and LC patients. The ROC curves were used to show the predictive ability.

To further validate the predictive capacity of sB7-H5 in GC, CRC, and LC patients, a ROC curve analysis was performed. A larger AUC indicates a higher diagnostic value, and AUC > 0.5 was considered to have a diagnostic value. As shown in Figure 5, the AUC value of sB7-H5 in GC, CRC, and LC patients was 0.6439, 0.5891, and 0.6587, respectively. These data suggested that sB7-H5 is a potential diagnostic marker of GC, CRC, and LC.

## 4. Discussion

Currently, cancer immunotherapy based on ICIs has been regarded as one of the most effective strategies in the treatment of various human cancers [6, 21]. As a crucial immune checkpoint molecule, B7-H5 has been demonstrated to exert suppressive effects on the immune system [14]. The study by Böger et al. showed that the expression of B7-H5 in the tissue samples of GC patients was associated with the Laurén phenotype, tumor localization, Epstein-Barr virus infection, KRAS-and PIK3CA-mutational status, and PD-L1 expression [9]. Furthermore, we have reported that upregulation of B7-H5 was significantly associated with lymph node involvement, American Joint Committee on Cancer (AJCC) stage, and survival of CRC patients. Blockade of B7-H5 could enhance the infiltration and Granzyme B production of CD8^+^ T cells and inhibit tumor growth in mouse tumor models [7]. In human non-small-cell lung cancer, B7-H5 was frequently overexpressed in the tumor tissues of patients and showed positive association with upregulated tumor-infiltrating lymphocytes, PD-1 axis markers, specific genomic alterations, and prognosis [10]. In the current study, we generated B7-H5 mAbs by immunizing mice with B7-H5 overexpressing L929 cells as previously described [19, 20]. The commercial B7-H5 antibody (D1L2G) was produced by immunizing animals with a synthetic peptide corresponding to residues near the carboxy terminus of human B7-H5 protein. The methods of B7-H5 mAb generation used in this study could produce more variety of B7-H5 antibodies. Additionally, we obtained 28 clones of mouse anti-human B7-H5 mAbs, which showed positive binding by ELISA. Among these clones, we screened two novel mouse anti-human B7-H5 mAbs 2E5 and 7B10 with different epitopes and established a sandwich ELISA system based on the two mAbs. Using this ELISA, we found that the concentration of sB7-H5 was markedly higher in GC, CRC, and LC patients than that in HCs, which indicated that the ectopic expression of B7-H5 is involved in the progression of multiple cancers including GC, CRC, and LC.

Recent evidence indicated that soluble B7-CD28 family inhibitory immune checkpoint molecules have been correlated with the progression of cancer and immunotherapy [15]. For example, the concentration of plasma soluble PD-1 was significantly increased in non-small-cell lung carcinoma, GC, and bladder cancer patients treated with anti-PD-1 antibody and correlated with tumor size progression [16]. The levels of serum soluble B7-H3 were upregulated in patients with GC and were positively associated with TNM stage or with infiltration depth T3/T4 or with lymph node metastasis [22]. In addition, it has been confirmed that soluble B7-H4 can be considered a diagnostic biomarker for multiple cancers, such as non-small-cell lung cancer, epithelial ovarian cancer, and GC [23–25]. Hence, it is worth exploring the expression of soluble immune checkpoint molecules in patients with cancer and assessing their predictive and prognostic value.

Interestingly, B7-H5 also exists in a soluble form, which is involved in the progression of the disease. Yasinska et al. showed that sB7-H5 released by acute myeloid leukemia cells could specifically bind galectin-9, block Tim-3-mediated signaling, and result in granzyme B-mediated self-killing of human T cells [26]. Serum CA19-9, PD-L1, PD-L2, and B7-H5 were markedly elevated in pancreatic cancer patients. And the combined detection (CA19-9+PD-L1+PD-L2+B7-H5) had a much higher sensitivity than the single CA19-9 detection [18]. Moreover, the levels of sB7-H5 were specifically upregulated in acute pancreatitis and functioned as a biomarker for diagnosis, severity, and prognosis [17]. Herein, our results noted that upregulation of sB7-H5 in the serum of patients with GC, CRC, and LC was associated with TNM stage and metastasis. Moreover, the diagnostic value of sB7-H5 in patients with GC, CRC, or LC was evaluated via ROC curve analysis. We found that the AUC value of sB7-H5 in GC, CRC, and LC patients was 0.6439, 0.5891, and 0.6587, respectively. Taken together, these results indicated that sB7-H5 may represent a potential biomarker for diagnosing of tumors and a target for cancer treatment.

The current study comprised 50 GC patients, 74 CRC patients, and 122 LC patients. Given that the sample size was relatively small, the significance of our study is limited. Further investigations are needed in a larger population to elucidate the expression pattern and clinical significance of sB7-H5 in GC, CRC, and LC. To our best knowledge, the roles and molecular mechanisms of sB7-H5 in regulating the progression and immune escape of cancer remain unclear. Hence, it may be valuable to explore the effect of sB7-H5-mediated biological function on tumor progression in our future study.

In summary, we developed two novel mouse anti-human B7-H5 mAbs, 2E5 and 7B10, and further established a novel sandwich ELISA for the detection of sB7-H5. Moreover, our results indicated that the serum sB7-H5 levels were significantly increased in patients with GC, CRC, and LC and positively associated with TNM stage and metastasis. Therefore, a diagnostic or therapeutic strategy based on sB7-H5 may be a promising approach for cancer treatment.

## Figures and Tables

**Table 1:** The relationship between the levels of sB7-H5 in sera of patients with GC and clinicopathological characteristics.

Characteristics	*N*	sB7-H5 expression		*U*	*P*
		Median (Min–Max)	*P*_25_ ~*P*_75_		
Age					
<68	24	421.0 (306.9-4512)	357.8-516.4	224.0	0.0890
≥68	26	388.2 (156.2-911.1)	265.4-511.0		
Gender					
Male	37	428.7 (156.2-1018)	331.6-518.9	204.0	0.4230
Female	13	369.5 (159.8-4512)	327.1-436.2		
TNM					
I+II	14	361.2 (194.6-508.5)	304.2-400.0	155.0	0.0353^∗^
III+IV	36	448.0 (156.2-4512)	342.5-527.7		
Metastasis					
No	13	381.5 (194.6-518.5)	321.4-403.8	165.0	0.0955
Yes	37	442.9 (156.2-4512)	334.2-526.1		

TNM: tumor node metastasis. ^∗^*P* < 0.05.

**Table 2:** The relationship between the levels of sB7-H5 in sera of patients with CRC and clinicopathological characteristics.

Characteristics	*N*	sB7-H5 expression		*U*	*P*
		Median (Min–Max)	*P*_25_ ~*P*_75_		
Age					
≤65	37	382.1 (157.4-715.0)	275.8-479.3	580.0	0.2614
>65	37	396.7 (168.2-1185)	311.4-510.0		
Gender					
Male	48	358.4 (169.4-1184)	288.1-497.8	614.0	0.9127
Female	26	406.7 (157.4-1185)	295.7-496.8		
TNM					
I+II	17	283.6 (159.8-607.4)	240.9-387.4	264.0	0.0040^∗∗^
III+IV	57	412.6 (157.4-1185)	322.5-510.0		
Metastasis					
No	14	282.4 (159.8-607.4)	218.1-401.6	236.0	0.0101^∗^
Yes	60	405.6 (157.4-1185)	321.5-504.4		

TNM: tumor node metastasis. ^∗^*P* < 0.05; ^∗∗^*P* < 0.01.

**Table 3:** The relationship between the levels of sB7-H5 in sera of patients with LC and clinicopathological characteristics.

Characteristics	*N*	sB7-H5 expression		*U*	*P*
		Median (Min–Max)	*P*_25_ ~*P*_75_		
Age					
≤67	62	389.2 (134.7-925.2)	338.2-453.6	1588	0.1646
>67	60	415.0 (213.8-1655)	360.5-527.9		
Gender					
Male	75	403.3 (216.2-1191)	359.8-470.2	1728	0.8576
Female	47	409.1 (134.7-1655)	327.0-492.5		
TNM					
I+II	32	388.4 (140.6-501.5)	273.1-453.7	1089	0.0405^∗^
III+IV	90	409.0 (134.7-1655)	360.3-505.0		
Metastasis					
No	22	325.6 (140.6-501.5)	247.9-428.7	629.5	0.0014^∗∗^
Yes	100	413.0 (134.7-1655)	360.5-486.7		

TNM: tumor node metastasis. ^∗^*P* < 0.05; ^∗∗^*P* < 0.01.

**Table 4:** Precision of the human sB7-H5 ELISA system.

Sample	Intra-assay precision			Interassay precision		
	600 pg/ml	300 pg/ml	150 pg/ml	600 pg/ml	300 pg/ml	150 pg/ml
*N*	10	10	10	10	10	10
$\bar{X}$	318.65	159.75	79.42	339.23	171.26	85.22
SD	10.02	9.48	4.848	22.53	11.713	6.96
CV	3.148	5.938	6.10	6.64	6.84	8.16

Abbreviations: ELISA: enzyme-linked immunosorbent assay; *N*: number; SD: standard deviation; CV: coefficient of variation.

## Data Availability

The data used to support the findings of this study are included within the article.
